# Erector spinae plane block versus caudal block in children: similar analgesia, different stories beneath the surface

**DOI:** 10.1016/j.bjane.2026.844759

**Published:** 2026-04-22

**Authors:** Tuhin Mistry, Abhijit Sukumaran Nair

**Affiliations:** a Ganga Medical Centre & Hospitals Pvt Ltd, Department of Anaesthesiology and Perioperative Care, Coimbatore, India; b Ibra Hospital, Department of Anaesthesiology, Ibra, Sultanate of Oman

Dear Editor,

We read with interest the systematic review and meta-analysis comparing Erector Spinae Plane Block (ESPB) with Caudal Epidural Block (CEB) in pediatric surgery.^1^ In a specialty where practice often reflects institutional preference rather than robust evidence, such attempts to systematically synthesize available data are especially valuable. We would like to offer a few comments that may help readers interpret the findings and guide future research.

First, there is some inconsistency in the reporting of evidence certainty. The abstract states that all outcomes were supported by high-quality evidence, whereas the main text and GRADE summary indicate very low certainty for the primary outcome (time to first rescue analgesia) and moderate certainty for FLACC scores at 24 hours, with high certainty applying solely to Postoperative Urinary Retention (POUR) and Postoperative Nausea and Vomiting (PONV). This discrepancy may mislead readers who rely primarily on the abstract. Clarifying it and emphasizing that the primary outcome is supported by low-certainty evidence due to marked heterogeneity and imprecision, would provide a more balanced representation of how confidently equivalence between ESPB and CEB can be inferred.

Second, the extremely high heterogeneity for the primary analgesic outcome (I^2^ = 99%) substantially limits the interpretability of a single pooled estimate. Persistence of heterogeneity suggests that multiple clinically meaningful effect modifiers are contributing: variations in patients’ age, inclusion of diverse surgical procedures (inguinal hernia, hypospadias, circumcision, hip and proximal femur surgery, renal surgery, lower limb surgery), variation in ESPB levels (sacral, lumbar, thoracic), and marked differences in local anesthetic concentrations and volumes. These factors may not only generate statistical heterogeneity but also question the clinical validity of aggregating all trials into a single summary measure. While subgroup analyses by surgical category or ESPB level could offer useful insights, the relatively small number of available trials would likely limit statistical power and precision. Such analyses should therefore be viewed as hypothesis-generating rather than definitive, but they may still help frame future research questions.

Third, ESPB is treated as a uniform technique, although sacral, lumbar, and thoracic ESPB represent anatomically and mechanistically distinct blocks.^2^ Sacral ESPB performed for procedures such as hypospadias or circumcision differs fundamentally from lumbar or lower thoracic ESPB used for hip or femur surgery, and these techniques may not be clinically interchangeable in terms of injectate spread, neural targets or analgesic profile. Therefore, pooling these under a single label risks masking level-specific outcomes. Future reviews may benefit from evaluating sacral ESPB separately or classifying it more precisely as a sacral multifidus plane block,^3^ particularly when the comparator is a sacral neuraxial block such as CEB.

Fourth, although the selected outcomes (time to first analgesic request and FLACC scores) are clinically relevant, they provide a limited view of postoperative recovery. Pediatric- and caregiver-centred metrics such as opioid consumption, block success rates, ease of performance, motor block, early mobilization, length of stay, and parent/patient satisfaction would enhance real-world clinical applicability. The authors’ focus on POUR is commendable, and the lower incidence observed with ESPB is clinically meaningful.^1^ However, POUR may be influenced by factors that are not consistently reported across trials, including neuraxial opioid use, catheterization practices, and the proportion of urological procedures.^4^ Clear reporting of these trial-level details would aid interpretation and support more nuanced patient selection. Finally, we appreciate the authors’ call for standardized protocols and detailed reporting in future RCTs. Building on this, future studies may consider prespecifying minimally important differences for pain scales, standardizing or at least stratifying local anesthetic regimens and adjuvants, and adopting core outcome sets that incorporate safety, functional recovery, and patient-reported outcomes. These refinements would improve methodological rigor and facilitate more definitive meta-analytic conclusions. Such efforts are crucial for determining when ESPB can be reliably considered an alternative to CEB, particularly in ambulatory pathways or in children at elevated risk for POUR.

In conclusion, this review provides a timely comparison of ESPB and CEB, indicating similar analgesic performance but highlighting potential benefits of ESPB for avoiding POUR. As pediatric regional anesthesia evolves, more anatomically specific study designs and standardized reporting will be essential for defining when ESPB can reliably replace caudal techniques.

## Data availability statement

No new data were created or analysed in this letter. Data sharing is not applicable to this article.

## Declaration of AI use

The authors confirm that no AI tools or Language Models (LLMs) were used in the writing or editing of the manuscript.

## Authors’ contributions

Tuhin Mistry: Conceptualization; methodology; writing-original draft; review & editing.

Abhijit Sukumaran Nair: Validation; writing-review & editing; supervision.

## Funding

No funding was received.

## Conflicts of interest

The authors declare no conflicts of interest.
